# Treatment envelope of transcranial histotripsy: challenges and strategies to maximize the treatment location profile

**DOI:** 10.1088/1361-6560/ad8d9f

**Published:** 2024-11-11

**Authors:** Ning Lu, Ellen M Yeats, Jonathan R Sukovich, Timothy L Hall, Aditya S Pandey, Zhen Xu

**Affiliations:** 1 Department of Radiology, Stanford University, Palo Alto, CA 94304, United States of America; 2 Department of Biomedical Engineering, University of Michigan, Ann Arbor, MI 48109, United States of America; 3 Department of Neurosurgery, University of Michigan, Ann Arbor, MI 48109, United States of America

**Keywords:** acoustic cavitation, histotripsy, phased array, transcranial brain therapy, treatment envelope

## Abstract

A 750 kHz, 360-element ultrasound array has been built for transcranial histotripsy applications. This study aims to evaluate its performance to determine whether this array is adequate for treating a wide range of brain locations through a human skull. Treatment location profiles in 2 excised human skulls were experimentally characterized based on passive cavitation mapping. Full-wave acoustic simulations were performed in 8 human skulls to analyze the ultrasound propagation at shallow targets in skulls with different properties. Results showed that histotripsy successfully generated cavitation from deep to shallow targets within 5 mm from the skull surface in the skull with high SDR and small thickness, whereas in the skull with low SDR and large thickness, the treatment envelope was limited up to 16 mm from the skull surface. Simulation results demonstrated that the treatment envelope was highly dependent on the skull acoustic properties. Pre-focal pressure hotspots were observed in both simulation and experiments when targeting near the skull. For each skull, the acoustic pressure loss increases significantly for shallow targets compared to central targets due to high attenuation, large incident angles, and pre-focal pressure hotspots. Strategies including array design optimization, pose optimization, and amplitude correction, are proposed to broaden the treatment envelope. This study identifies the capabilities and limitations of the 360-element transcranial histotripsy array and suggests strategies for designing the next-generation transcranial histotripsy array to expand the treatment location profile for a future clinical trial.

## 1. Introduction

Brain pathologies such as brain tumors and intracerebral hemorrhage (ICH) can develop in any part of the brain ranging from central cerebral areas to cortex locations close to the skull interior surface (Larjavaara *et al* 2007), (Sun *et al* 2020), (An *et al* 2017). For example, gliomas were mostly observed in the cerebral cortex and only 14% were observed in the deeper structures (Larjavaara *et al* 2007); however, metastases can also occur in any region of the brain. Meningiomas form along the dura and consequently tend to occur near the surface of the brain or skull base (Sun *et al* 2020). In addition, ICH commonly occurs in both superficial cerebral vessels and deep central structures such as the thalamus, basal ganglia, putamen, etc (An *et al* 2017), (Nichols *et al* 2021). Therefore, the ideal goal for noninvasive focused ultrasound (FUS) therapy is to treat varied brain locations including deep, superficial, anterior, and posterior to allow for the greatest patient care translation. Current commercial transcranial MR-guided focused ultrasound (TcMRgFUS) brain systems can only treat deep target locations in the central region of the brain due to the risks of overheating on the skull, scalp, and brain surface (Hynynen 1990), (Martin *et al* 2009), (McDannold *et al* 2010), (Arvanitis *et al* 2016). The treatment location envelope near the skull base has been investigated, demonstrating the closest treatable distance of 17.4 ± 1.9 mm from the skull base without skull overheating (Pulkkinen *et al* 2011). However, safe treatment at such a distance in the cerebral cortex has only been modeled numerically for TcMRgFUS but has not been shown experimentally (Pichardo and Hynynen 2007). Depending on the geometry and acoustic properties of the skull, the absorbed heat within the skull may lead to bone marrow damage following TcMRgFUS (Schwartz *et al* 2018). In recent TcMRgFUS clinical trials, bone marrow lesions were observed in 16 out of 40 treatments at 3–15 months post-treatment in MRI, where acoustic simulations also showed heated regions similar to the observed lesions in terms of locations, sizes, and shapes (McDannold *et al* 2020). Additionally, the long burst pulses used in TcMRgFUS can produce standing waves near the skull that may cause hemorrhage during transcranial thrombolysis (Daffertshofer *et al* 2005, Deffieux and Konofagou 2010, Tang and Clement 2010, Song *et al* 2012). These safety concerns significantly constrain the feasible treatment location profile of tcMRgFUS, especially hindering the treatment of shallow locations close to the skull.

Large variations in skull acoustic properties have also been shown to highly impact the efficacy of FUS treatment, limiting the population of patients to whom FUS therapies can be effectively applied. Patients in TcMRgFUS clinical trials are screened using a metric known as the skull density ratio (SDR), defined as the ratio between the averaged value of radio density in CT Hounsfield units (HU) of trabecular bone in the middle plate of the skull to the cortical bone (Chang *et al* 2016 Meng *et al* 2020). SDR values close to 1 suggest the skull is weakly heterogeneous, whereas SDR values close to 0 indicate large variations are observed inside the skull, which may cause more transmission loss. An SDR of 0.4–0.5 was initially used as a range limit to determine candidacy for tcMRgFUS, which only covered ∼1/3 of the patient population (Jones *et al* 2020 Study Record 2023). Since these initial studies, patients with SDRs outside this range have also been treated based on medical needs (Boutet *et al* 2019 D’Souza *et al* 2019). Although SDR is currently used to screen patients for tcMRgFUS therapies, it is not always a reliable predictor of whether a given treatment will or will not be effective. Besides, SDR is usually calculated from clinical CT which does not resolve the skull microstructure. Attempts have been made to develop other metrics for TcMRgFUS patient screening (e.g. the Beam Index Sammartino *et al* 2019), but their clinical relevance in the context of transcranial histotripsy remains unclear. Additionally, acoustic properties at different anatomical segments on the same skull have been shown to vary substantially (Riis *et al* 2022). The effective SDR along the acoustic path is anticipated to increase as the target locations move from the central brain to near the skull surface, as the acoustic beams from transducer elements of the hemispherical array travel through a longer path in the trabecular layer at high incident angles.

Unlike TcMRgFUS, transcranial MR-guided histotripsy uses 1-cycle pulses with a very low duty cycle (<0.1%) to avoid overheating to the skull or surrounding tissue (Lin *et al* 2014, Zhang *et al* 2015, Gerhardson *et al* 2017). Governed by a threshold mechanism, histotripsy only generates cavitation where the rarefactional pressure amplitude exceeds an intrinsic threshold of 26 MPa. Besides, histotripsy uses a very short pulse length and a large-aperture array, both of which reduce the likelihood of wave constructive interference at a pre-focal location when reflected and have been shown to eliminate standing waves that are present when using long ultrasound pulses or continuous waves (O’Reilly *et al* 2010 Martin *et al* 2021). Previously, Sukovich *et al* successfully generated lesions in a tissue-mimicking phantom at 6–12 mm from the interior skull surface in one *ex vivo* human skull (2016). This showed the proof-of-concept of treating shallow targets near the skull surface using transcranial histotripsy. However, it is yet unknown whether such capability can be generalized for all patients with various skull properties using one transducer array.

Recently, a 750 kHz 360-element transcranial histotripsy human prototype system has been built by our group. One major design goal is to overcome the treatment location limitation of the tcMRgFUS thermal ablation to enable the treatment of various brain locations through the skull. This paper evaluates the performance of this transcranial array to determine whether the array is adequate to treat a wide range of locations through the human skull. We first investigate the treatment location profiles of transcranial histotripsy in two excised human skulls experimentally using the 360-element array. Then, we investigate the treatment location profile of this array in 8 human skulls with different acoustic properties by acoustic simulation. The results are used to identify the capabilities and limitations of the current 360-element design and propose potential improvements for the next-generation array to expand the treatment envelope of transcranial histotripsy in a broad patient population.

## 2. Methods

### 2.1. Histotripsy system

**Figure 1.**
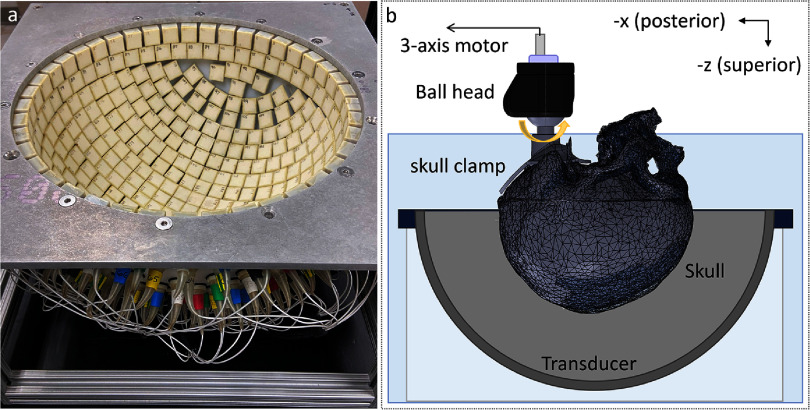
Photo of the transducer array (a) and the experimental setup (b) used in this study. The whole setup was submerged in degassed de-ionized water during experiments.

A 360-element, 750 kHz hemispherical phased array transducer with a radius of 15 cm was constructed for transcranial histotripsy treatment (figure 1(a)). The transducer modules were 17 mm squares fabricated with flat piezoceramic material (PZ36, CTS-Ferroperm, Kvistgaard, Denmark), 3D-printed housings, and matching layers, and installed into the array scaffold. Driving electronics were built in-house to generate 1-cycle, high-voltage pulses to drive the transducer modules. Compared to the commercial MRgFUS system with a rated driving voltage of 200–500 V (2016), our circuitry can generate a peak driving voltage of 3 kV, at which a single module can produce a peak-negative pressure of up to 1.5 MPa at a distance of 150 mm in the free field. Receiving circuity was also integrated with a 12-bit digitizer (AFE5801, Texas Instruments, Dallas, TX) to digitize the received acoustic signals with a high sensitivity and wide dynamic range. The transducer modules and transmit-receive functionality were controlled by system-on-a-chip FPGA devices.

### 2.2. Experimental evaluation of the treatment envelope

#### 2.2.1. Skull samples

Two excised human skulls purchased from an external vendor (Skulls Unlimited, Oklahoma City, OK, USA) were used to evaluate the treatment envelope experimentally. The skulls were stored in refrigerated de-ionized water and degassed for at least 12 h in a vacuum chamber before all experiments to minimize gas in the trabecular bone. The dimensions and acoustic properties of these skull samples are shown in table 1 (skulls #1 and #2), representing high and low SDRs.

**Table 1.** Measurements of the skulls from CT images.

No.	Dimension^a^ (mm)	Thickness^b^ (mm)	Skull density ratio (sdr)
1	179 *×* 142 *×* 155	4.9 ± 1.2	0.71
2	177 *×* 139 *×* 172	6.5 ± 1.7	0.35
3^*^	197 *×* 147 *×* 104	6.9 ± 1.7	0.52
4	185 *×* 142 *×* 194	8.7 ± 2.0	0.48
5	193 *×* 144 *×* 201	6.8 ± 1.5	0.48
6	209 *×* 146 *×* 160	7.0 ± 1.2	0.59
7	184 *×* 130 *×* 151	7.0 ± 2.4	0.71
8	182 *×* 149 *×* 157	7.1 ± 1.8	0.51

^a^ Measurements of the skulls’ dimensions were obtained from CT scans.

^b^ Mean ± standard deviation of the skull thickness.

^*^ Skull#3 is an excised skull calvarium, whereas other skull samples are full skulls, so the superior-to-inferior dimension of skull#3 is noticeably smaller compared to the others.

#### 2.2.2. Experimental setup

**Figure 2.**
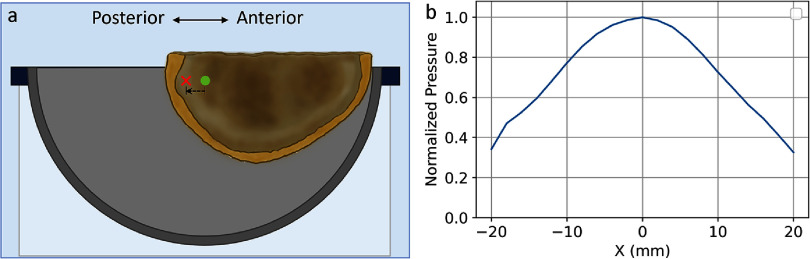
(a) Diagram of an example target with additional electronic focal steering. The desired target location is labeled as a red x, and the geometric focus of the array is labeled as a green dot. The skull cannot be placed to align the target location directly with the geometric focus due to sizes of the skull and array. Therefore, the skull is placed as shown in the diagram, and the array was electronically steered 10 mm (black dashed arrow) towards the posterior direction to focus on the target. (b) Electronic steering profile of the array through the skull on the anterior–posterior axis. Pressure amplitudes were measured with hydrophone-based aberration correction applied at each location and normalized to the amplitude at the geometric focus of the array.

During experiments, the skulls were affixed to a 3D-printed clamp mounted on a Ball Head (RRS, Lehi, UT, USA) and placed in the transducer array (figure 1(b)). Both the array and the skull were submerged in degassed de-ionized water during the experiments. The Ball Head was attached to a 3-axis motorized positioner, allowing targeting at various distances from the skull surfaces. For each skull, 34 anatomical target locations were tested, ranging from the deep center of the skull to within 1 cm from the skull interior surface in the anterior, posterior, superior, and lateral regions. The skull’s orientation was fixed in the array during the experiments, with the ear-to-ear cut plane approximately parallel to the equatorial plane of the hemispherical array. The skulls were moved translationally such that the geometric focus of the array was aligned with (or at least as close as possible to) the target locations (i.e. mechanical steering by moving the skull instead of moving the array location). For some targets in anterior and posterior regions, mechanical steering was not sufficient because the accessible range was limited by the skull dimension and the array geometry (figure 2(a)). In such cases, electronic focal steering was applied along the anterior-posterior axis up to 15 mm in addition to mechanical steering to reach the target location. It should be noted that the acoustic pressure decreases when the array is electronically steered away from the geometric focus, as shown in figure 2(b). The electronic focal steering is thus less preferable than the mechanical steering but necessary when mechanical steering cannot provide direct access to the desired target location.

#### 2.2.3. Aberration correction (AC)

The 2-step AC was implemented whenever possible to maximize pressure recovery, with CT-AC as the first step, followed by the cavitation-based AC using acoustic cavitation emission (ACE) signal as previously described in Lu *et al* (2022). At each target location, we first co-register the skull to the device by aligning the skull surface map reconstructed from acoustic pulse-echo data to the skull surface profile acquired from CT scans. The CT-based analytical correction was performed in an open-source toolbox, Kranion, which implements ray-tracing and calculates the phase difference across the elements (Sammartino *et al* 2019). The calculated delays from Kranion were applied as firing offsets with the driving voltage gradually ramping up till cavitation was generated. Then, the ACE shockwaves were recorded by the transducer array and used to refine the time delays using cross-correlation. At some target locations, cavitation could not be generated even with the maximal driving voltage. In such cases, the cavitation-based AC could not be implemented. Amplitude correction was not applied, i.e. all transducers were driven at the same amplitude.

#### 2.2.4. Cavitation generation, detection, and localization

The transducer array was excited at a pulse repetition frequency (PRF) of 10 Hz. Ten pulses were delivered to each target location. For each pulse, all transducer modules recorded waveforms at a sampling frequency of 12.5 MHz.

To characterize the treatment envelope, a binary cavitation detection was first performed to determine whether any cavitation shockwaves were observed in the received waveforms for each target. Because the ultrasound waves only propagated through the skull and degassed water and no nucleation agent such as microbubble was involved, the presence of cavitation shockwaves indicated the acoustic pressure through the skull was above the cavitation intrinsic threshold (∼26 MPa) (Haskell *et al* 2023). The output power was gradually ramped up until an ACE shockwave from bubble collapse was observed in the recorded waveforms from the transducers. The signal processing workflow to extract the ACE shockwaves followed the method described previously (Lu *et al* 2022). The received signals were shifted in phase against the inverse of the summed transmit and aberration correction delays. If no ACE event was observed in the shifted signal even at the maximal output, or an ACE event was observed but the bubble lifespan (the time duration between bubble nucleation and collapse) was less than 30 *µ*s, it suggested that cavitation could not be generated reliably, and the corresponding target location was categorized as ‘outside of the treatment envelope’.

For target locations where cavitation shockwaves were detected, we performed cavitation localization using the extracted shockwaves. Each ACE collapse signal was cropped in a time window with a duration ⩽8 *µ*s and individually processed using passive acoustic mapping (PAM). PAM localization utilizes coherent beamforming methods to identify the spatial location of an acoustic source by back-projecting acquired signals into the field, summing them together at each location, and identifying the position where the summed signal intensity is largest (Gyöngy *et al* 2008, Arvanitis *et al* 2017, Haworth *et al* 2017, Lu *et al* 2018, Gray and Coussios 2018). Since the ACE collapse only spanned a very short time window, the PAM results should not be prone to artifacts or noises from backscatter and reflections. Therefore, the standard delay-and-sum was implemented for PAM to save computational cost and time (Salgaonkar *et al* 2009), (Coviello *et al* 2015). For each target location, a profile of the skull exterior surface was reconstructed from pulse-echo waveforms as described previously (Lu *et al* 2022) and displayed on the 2D cross-section pressure amplitude map generated by PAM for cavitation localization. If the localized cavitation was within a 2 mm radius from the target location, the corresponding target location was categorized as ‘within the treatment envelope’. However, if any of the localized cavitation was outside of the target zone and in the pre-focal direction, the corresponding target location was labeled as ‘pre-focal cavitation detected.’

### 2.3. Acoustic simulation

#### 2.3.1. Skull dataset

To explore a greater diversity of skull properties, we performed acoustic simulations with all 8 human skulls listed in table 1. All human skulls were acquired through the Anatomical Donation Program at our institution, following the same protocol as previously described (Lu *et al* 2022). The skulls were imaged with a clinical CT scanner (Discovery CT 750 HD, GE Healthcare, USA) at a voltage of 120 kVp, with an in-plane resolution of 0.488 × 0.488 mm^2^, and a slice thickness of 1.25 mm (reconstructed to 0.625 mm by the built-in reconstruction kernel ‘BONE+’). The acoustic properties of the 8 skulls are summarized in table 1. The SDR of these skull samples spanned from 0.3 to 0.7, and their mean thicknesses varied from 4.9 to 8.7 mm.

**Figure 3.**
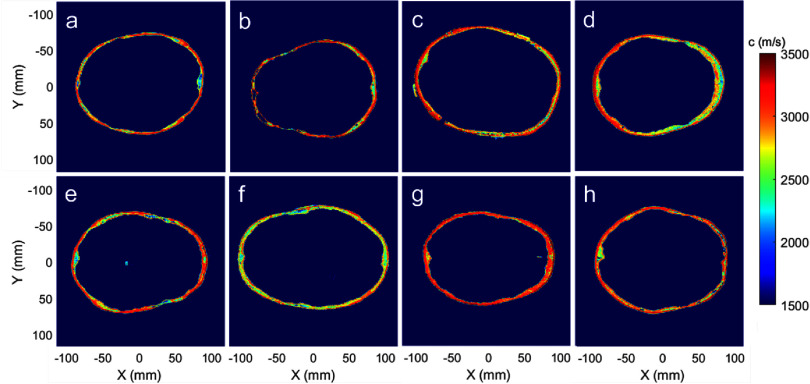
Cross-section of the sound speed map for 8 skulls used in acoustic simulation with target location at the center of the skull. (a)–(h): Skull #1–#8. The spatial resolution of the sound speed maps is 0.5 mm, determined by the resolution of k-Wave simulation and the resolution of CT scans.

The skull volumes were segmented in MATLAB by thresholding the HU values. Their sound speed ${c_s}$ and density ${\rho _s}$ were estimated using Marsac’s mapping (${\text{H}}{{\text{U}}_{\min }}$ = 1050, ${\text{H}}{{\text{U}}_{\max }}$ = 3100, ${\rho _{\min }}$ = 1000 kg m^−3^, ${\rho _{\max }}$ = 2500 kg m^−3^, ${c_{\min }}$ = 1482 m s^−1^, ${c_{\max }}$ = 3500 m s^−1^) (Marsac *et al* 2017). Figure 3 shows example sound speed maps on the focal plane for all 8 skulls when targeting the center of the skull, and demonstrates the large variation in shape, size, and acoustic properties among tested skull samples. It should be noted that the microstructures in human trabecular bone have an element size of 40–150 *µ*m with a spacing of 0.5–1.5 mm, therefore, the porous microstructures could not be resolved at the resolution of the clinical CT system (Chaffai *et al* 2002 Aubry *et al* 2003 Pinton *et al* 2012).

### 2.4. Transducer element model

**Figure 4.**
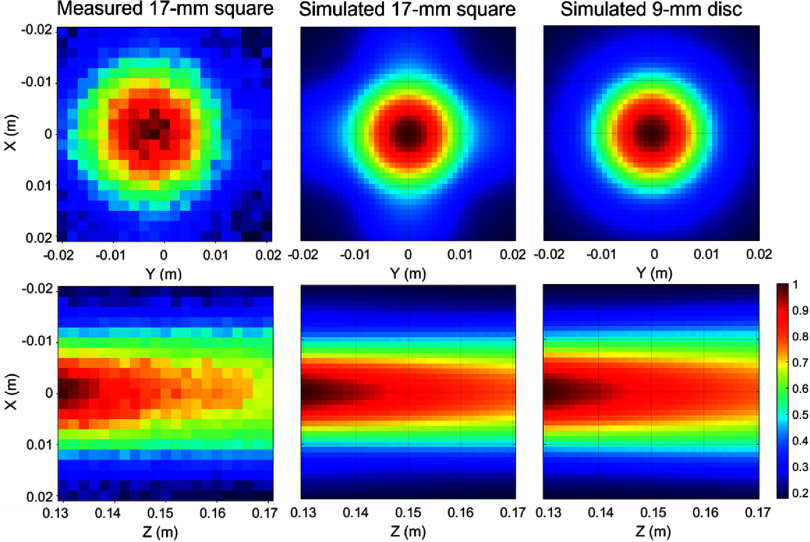
Comparison of the measured and simulated beam patterns at the focal distance of 150 mm. Left: Beam pattern of the 17 mm square element measured by fiber-optic hydrophone. Middle: Simulated beam pattern of a 17 mm square element on lateral and axial planes. Right: Simulated beam pattern of a round element with radius = 9 mm on lateral (*X–Y*) and axial planes.

Previous studies have indicated that square-shaped or polygon-shaped transducers constructed from solid piezoelectric materials typically produce an acoustic field similar to that of a round transducer, with high-pressure amplitude at the center and often very small pressure amplitudes around the corners (Stocker *et al* 2022). To accurately model the square transducer elements in simulation, we simulated the output from square and round transducers with different sizes in FOCUS Toolbox (www.egr.msu.edu/∼fultras-web/). The simulated pressure fields were compared to the beam pattern of a 17 mm square element measured by a calibrated fiber-optic hydrophone at a focal distance of 150 mm from the surface of the transducer. Figure 4 shows that the measured beam profiles were matched most closely in simulation by a disc transducer with a radius of 9 mm. Therefore, each transducer element on the hemispherical array was modeled as a disc with a 9 mm radius in the following simulations.

### 2.5. Attenuation at deep versus shallow targets

The suitability of using a linear acoustic propagation model for simulating histotripsy attenuation and aberration effects has been investigated previously (Yeats *et al* 2022). Acoustic simulations were performed to model ultrasound propagation through 8 skulls at various target locations using k-Wave, an open-source MATLAB toolbox (Treeby and Cox 2010). To investigate how transcranial attenuation changes with target location, we performed simulations at 9 target locations for each skull: one deep central target (C) (∼60 mm from the skull interior surface), 4 shallow targets that are 10 mm from the skull surface in the anterior (A), posterior (P), superior (S), and lateral (L) regions, and 4 intermediate—shallow targets that were 20 mm from the skull surface in the A, P, S, and L regions. As previously shown, the deep central location is easier to target compared to shallow targets and the currently ongoing clinical studies of tcMRgFUS are mostly targeting the deep brain (Franzini *et al* 2020 Elias *et al* 2016). The shallow targets were set at 10 and 20 mm from the skull surface in the cerebral cortex lobes because a high occurrence of brain tumors and strokes has been reported at these locations in human patients (Larjavaara *et al* 2007 Sun *et al* 2020 Nichols *et al* 2021). One free-field simulation was performed to provide a baseline pressure amplitude for calculating the focal pressure loss through the skull.

In all transcranial simulations, the skulls were oriented the same as they were placed experimentally in the array, with the ear-to-ear cut plane approximately parallel to the equatorial plane of the hemispherical array. For each target, the skull was positioned so that the target aligned with the geometric focus of the transducer array. Thus, the simulation did not involve any pressure loss due to the electronic focal steering.

For each target, 3 simulations were performed. The first simulation modeled reverse propagation by placing a point source at the target and receiving at the simulation grid point closest to the center of each transducer element. The received waveforms were cross-correlated to a reference channel to determine the differences in arrival time across transducer elements. Relative delays were calculated by subtracting the minimum arrival time from each element’s arrival time. The other two simulations modeled forward propagation using the 9 mm radius disc elements described above and recorded the maximum pressure amplitude over the entire acoustic field. In the first forward simulation, all transducers transmitted a 1-cycle 750 kHz Gaussian waveform with 50% bandwidth synchronously, thus modeling focusing without AC. In the second forward simulation, the pulse was transmitted from each transducer element with phase delays to compensate for the relative arrival time differences acquired using the reverse propagation simulation. This simulation modeled an ideal time-reversal-based phase correction through the skull, where the transducer surface is composed of point elements with sub-wavelength spacing and the arrival delay for each element is known exactly. All transducers were driven at the same amplitude in the forward simulations.

The attenuation coefficients ${\alpha _{{\text{skull}}}}$ and ${\alpha _{{\text{brain}}}}$ were set to 8.8 and 1.2 dB/(MHz^y^ cm) for skull and brain voxels, respectively, with a power law exponent *y* = 2 (IT’IS Foundation 2023). The sound speed and density of the skull were estimated as described in section II.C.1 above. The sound speed and density at the non-skull voxels were set to 1482.5 m s^−1^ and 998.2 kg m^−3^, respectively, calculated by the k-Wave functions *waterSoundSpeed* and *waterDensity* at 20 °C. The elastic wave propagation was not modeled in this study. A Courant–Friedrichs–Lewy number of 0.15 was used to ensure the stability of the simulation, providing a spatial step of 0.5 mm and a time step of 21.4 ns. The simulation domain spanned 320 ×320 ×216 mm (640 ×640 ×432 grid points) and a time duration of 180 *µ*s (8403 timesteps). All simulations included an 8 mm artificial absorbing layer at the edges of the simulation grid to prevent wrapping artifacts due to periodic Fourier boundary conditions. Simulations were accelerated by a distributed computing cluster. Each simulation was parallelized over 36 computing cores (Xeon Gold 6154 3.00 GHz, Intel, Santa Clara, California, USA) with 70 GB of collective memory. The average simulation execution time was 3 h.

### 2.6. Pressure contribution by individual transducers

Previous studies have shown that large incident angles significantly reduce the acoustic transmission at the tissue-skull interfaces due to a combination of refraction and mode conversion (Hayner and Hynynen 2001, Sammartino *et al* 2019, Bancel *et al* 2021). Ray-tracing showed that for targets close to the skull surface, the incidence angles of most array elements with respect to the skull surface were large and/or beyond the critical angle. Therefore, in addition to analyzing the overall attenuation and aberration effects, it is also important to understand how acoustic beams from individual transducers propagate through the skull and how much they each contribute to the focal pressure at shallow targets. Case studies were performed at a superior target 10 mm from the skull surface in skulls #2 and #5 by simulating the acoustic propagation from each transducer element separately through the skull (360 simulations per case). The metrics used to quantify the propagation performance from each transducer were defined as follows:

1. Focal pressure amplitude ${P_{{\text{focal}}}}$: the maximal pressure amplitude within the focal zone (±5 mm cubic volume centered at the target location).
2. Pre-focal hotspot pressure amplitude ${P_{{\text{hotspot}}}}$: If the pressure amplitude is observed to be higher at a location on the skull surface or within the skull than at the intended focal location, we define that pre-focal location with high amplitude as a pre-focal hotspot. ${P_{{\text{hotspot}}}}$ denotes the maximal pressure amplitude within the pre-focal hotspot zone (±2 mm cubic volume centered at the pre-focal hotspot. If more than one pre-focal hotspot is observed, the volume of interest is centered at the pre-focal hotspot with the largest pressure amplitude).

To understand the correlation between incident angles and the pressure contribution from individual elements, ray-tracing was implemented in Kranion for these two cases to estimate the incident angle at these target locations correspondingly.

## 3. Results

### 3.1. Experimental results

#### 3.1.1. Treatment location profile

**Figure 5.**
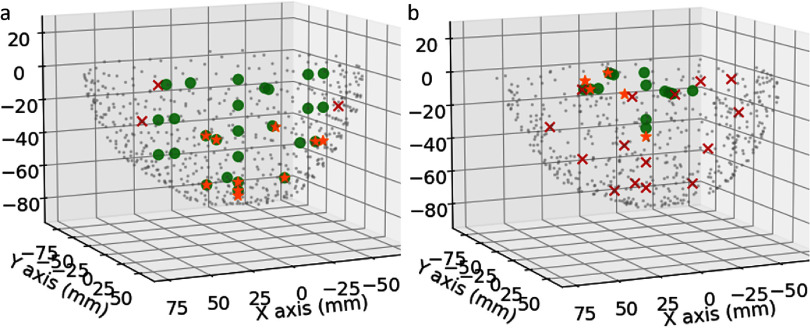
Treatment envelope indicated by ACE detection in skull #1 (a) and skull #2 (b), respectively. Grey dots: vertices on the skull outer surface mesh reconstructed from CT scans. Green dot: on-target cavitation detected; orange star: pre-focal cavitation detected; red X: no cavitation events.

In Skull #1 (SDR = 0.71; average thickness = 4.9 mm), the histotripsy system was able to generate cavitation confirmed by ACE signals at locations from the center to up to ⩽5 mm from the skull interior surface in the superior region and 10 mm in anterior, posterior, and lateral regions (figure 5(a)). However, in Skull #2 (SDR = 0.35; average thickness = 6.5 mm), cavitation could only be generated up to 16 mm and 35 mm from the skull surface in lateral and anterior regions, respectively (figure 5(b)). Cavitation events were not observed at locations <40 mm from the skull surface in posterior and superior regions in skull #2. These results suggested that the global and local acoustic properties of skulls played an important role in determining the treatment envelope, i.e. a thin skull with a high SDR (skull #1) was shown to be accessible by histotripsy treatment in a very wide range of locations, while a skull with a lower SDR and larger average thickness (skull #2) resulted in a more acoustic attenuation, and hindered effective treatment of shallow targets to varying degrees depending on their position.

### 3.2. Pre-focal cavitation

**Figure 6.**
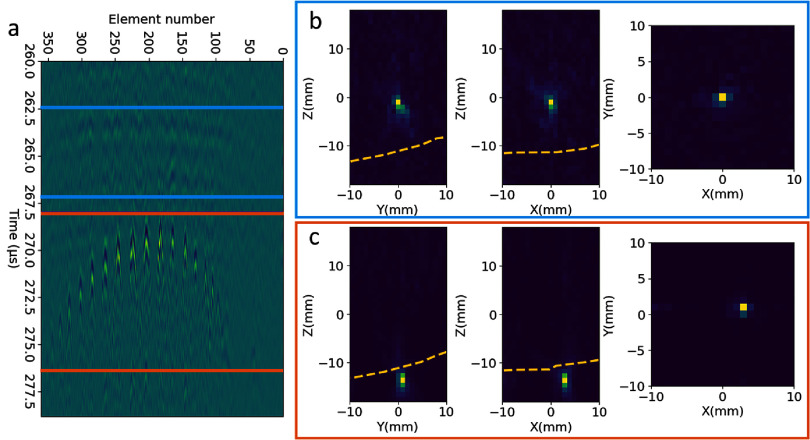
(a) Representative shockwaves from a single therapeutic pulse generating two cavitation events collapsed at different locations in space and time when targeting 5 mm from the skull interior surface at a superior location. Blue: on-target cavitation; Orange: pre-focal cavitation. (b) Corresponding time-resolved cavitation map of the on-target cavitation. (c) Corresponding time-resolved cavitation map of the pre-focal cavitation. Focus at [0,0,0], -*z* = pre-focal direction. The yellow dashed line indicates the skull’s exterior surface.

In both skull #1 and #2, pre-focal cavitation events were more likely to occur on or near the skull surfaces when targeting close to the skull surface compared to central locations. In skull #1, pre-focal cavitation was not observed for targets that are as close as 12 mm from the skull surface in all 4 regions (figure 5(a)). When targeting in the range of 5–12 mm from the skull surface in superior and lateral regions, pre-focal cavitation and on-target cavitation were both nucleated during singular firings of the array but collapsed at slightly different times after the firing. Figure 6 shows example ACE signals from a single histotripsy pulse where two cavitation bubbles, one on-target and the other 2 mm from the exterior surface of the skull in the pre-focal direction, were generated. The on-target cavitation collapsed at 263 *µ*s, whereas the pre-focal cavitation collapsed at around 270 *µ*s post array firing. In some cases, multiple, spatially distinct pre-focal cavitation events were observed through skull #1 as well as on-target cavitation during single firings of the array.

However, in skull #2, on-target cavitation was not generated at any target locations where the pre-focal cavitation was observed, suggesting that the generation of pre-focal cavitation events is likely related to the skull properties and may prevent focal cavitation. As shown in table 1 and figure 3, skull#2 has a larger skull thickness, lower SDR value, and larger variance of sound speed, which cause more reflection, scattering, and mode conversion inside the skull. Figure 3 also illustrates that skull#2 is less hemispherical and has more ridges than skull#1, which can result in shear wave conversion due to higher incident angles on the skull surface. All these skull properties are possible reasons for high attenuation and pre-focal cavitation, and the pre-focal cavitation can detract from and block the acoustic propagation to the target location.

### 3.3. Simulation results

#### 3.3.1. Attenuation at deep versus shallow targets

Table 2 summarizes the acoustic simulation results at various target locations. In all skulls, the transcranial focal pressure loss at the central target ranged from 70.0% to 85.8% with no AC and was reduced to 45.1% to 58.0% when the ideal time-reversal AC was applied. However, for shallow targets that are 10 mm from the skull interior surface, the focal pressure loss increased substantially to up to 95.8% due to the increases in incident angles and the elongation of the acoustic path through the skull due to refraction. The ideal AC recovered only <6% of lost focal amplitude at shallow targets, suggesting that focal pressure losses result from a combination of high incidence angles, increased attenuation losses due to increases in the acoustic path length, and aberrations when targeting close to the skull surface. High incidence angles can also cause shear wave conversion (not modeled here), which would further limit focal pressure at shallow targets.

**Table 2.** Summary of simulation results for 8 human skull samples.

No.	Focal pressure loss at central target (%)		Focal pressure loss at 20 mm to skull surface (%)		Focal pressure loss at 10 mm to skull surface (%)		Fraction of targets where pre-focal hotspots are observed			
							No AC		AC	
	No AC	AC	No AC	AC	No AC	AC	20 mm	10 mm	20 mm	10 mm
1	70.0	45.1	86.4–92.1	79.8–87.5	89.1–93.4	84.8–88.9	0/4	3/4	0/4	1/4
2	82.8	57.1	92.0–95.5	84.1–90.2	93.9–96.2	88.5–93.3	4/4	4/4		1/4
3^*^	85.8	56.4	86.5–93.2	80.8–90.2	92.4–94.5	89.6–91.3	2/4	4/4		1/4
4	82.9	54.3	91.9–94.2	84.2–88.9	93.5–95.8	89.3–92.0	3/4	4/4		2/4
5	81.1	57.6	88.0–95.0	82.6–89.6	91.2–95.8	85.9–91.3	3/4	4/4		1/4
6	74.6	51.3	88.0–91.3	81.2–84.1	90.4–93.8	87.0–87.3	0/4	4/4		0/4
7	83.8	58.0	90.2–95.7	84.2–86.8	92.0–95.6	88.4–93.2	3/4	4/4		3/4
8	80.8	56.8	89.0–94.2	85.0–90.0	90.4–95.7	88.0–95.1	3/4	4/4		2/4

Without aberration correction, pre-focal hotspots were observed at almost all shallow targets that were 10 mm from the skull surface in all 8 skull samples, except in the anterior region in skull #1 (table 2). In 6 out of 8 skull samples, pre-focal hotspots were also observed at half or more targets that were 20 mm from the skull surface. In general, the probability of pre-focal cavitation increased with proximity to the skull. However, the minimum target-to-skull distance at which pre-focal hotspots are observed varied depending on both the target region and the acoustic properties of the skull.

**Figure 7.**
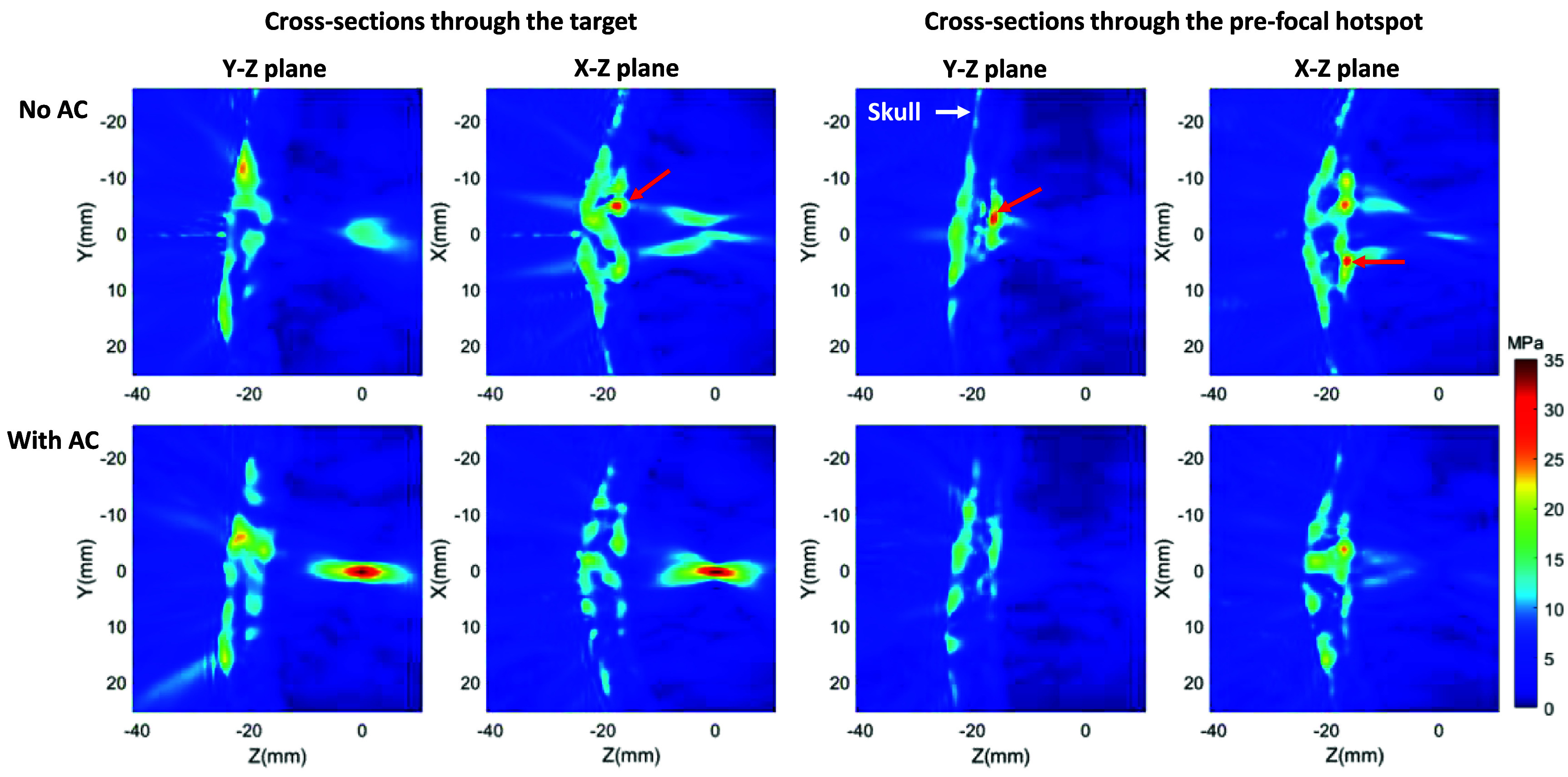
Simulated pressure field maps for superior target 10 mm from skull interior surface in skull #2. Indented target at [0,0,0]. Top row: No AC; bottom row: with AC. White arrow: skull. Red arrow: pre-focal pressure hotspots. Amplitudes in all maps were scaled to a total pressure output of 300 MPa from the array using the pressure loss ratio through the skull calculated from the simulation.

Aberration correction significantly reduced the pressure amplitude of the pre-focal hotspot and increased the focal pressure. As summarized in table 2, when ideal aberration correction was applied, pre-focal hotspots were eliminated in all skulls when the target location was 20 mm from the skull surface. For targets that were 10 mm from the skull surface, the fraction of targets where pre-focal hotspots occurred was largely reduced too. Figure 7 shows example simulated pressure maps for a superior target 10 mm from the skull surface in skull #2. Without AC, multiple pre-focal pressure hotspots were observed on the skull surface, among which the largest pressure amplitude was estimated to be 30.5 MPa and located at [5.0, −2.5, −16.0]. The focal pressure field at the intended target [0, 0, 0] was significantly distorted, resulting in a focal pressure amplitude <60% of the pre-focal pressure amplitude. When ideal time-reversal AC was applied, the focal pressure field was successfully refocused to provide a maximal amplitude of 34.8 MPa at the intended target. Besides, the pre-focal pressure amplitudes were significantly reduced to below the intrinsic cavitation threshold.

### 3.4. Pressure contribution by individual transducers

**Figure 8.**
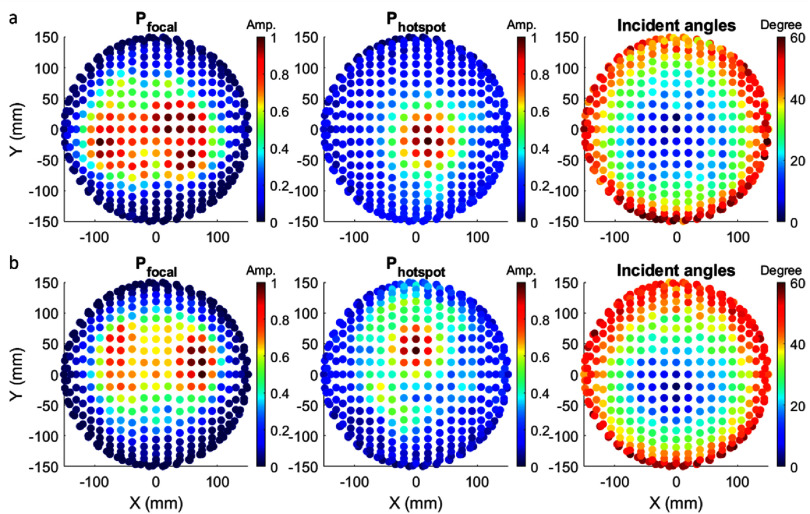
Pressure contribution by individual transducers in skull #2 (a) and skull #5 (b) at a superior target 10 mm from the skull surface. Left: Maximum pressure within the focal zone ${P_{{\text{focal}}}}$ generated by individual transducer elements. Middle: ${P_{{\text{hotspot}}}}$ contributed by individual transducer elements to the pre-focal hotspot. Right: Incident angles on the skull exterior surface from the individual transducer elements.

For the superior target 10 mm from the skull surface, the pressure at both the focus and the pre-focal hotspot resulted primarily from a small number of elements on the hemispherical array (figure 8). For example, 50% of the pressure at the hotspots was attributed to only 16% and 24% of the array elements in skull #2 and skull #5, respectively. The subset of elements that contributed most to ${P_{{\text{focal}}}}$ and ${P_{{\text{hotspot}}}}$ were in the sub-aperture of array elements with incident angles ⩽25°. Furthermore, the number of elements primarily contributing to the pre-focal pressure hotspot was significantly less than those contributing to the pressure in the focal zone. Incident angles >25° were observed on the outer rim of the hemispherical array, but those elements did not contribute much to either the focal pressure or pre-focal hotspot because their acoustic beams were largely scattered and reflected upon incidence on the skull exterior surface.

## 4. Discussion

This study evaluates the treatment location profile of transcranial histotripsy using a 360-element hemispherical transducer array prototype. Treatment location profiles in 2 excised human skulls were first experimentally characterized based on passive cavitation mapping. The experimental results demonstrated that the prototype system can generate cavitation as close as 12 mm to the skull surface without causing pre-focal pressure hotspots in selected skulls for transcranial histotripsy. To evaluate the feasibility of using this prototype system for a broader group of patients, we performed full-wave acoustic simulations in 8 human skulls with varying acoustic properties. Results indicate that the prototype system has limitations in treating shallow targets due to attenuation and pre-focal cavitation. It is possible to expand the treatment envelope for transcranial histotripsy, i.e. increasing the focal pressure above the cavitation threshold while maintaining the pressure outside the focus below the threshold, by improving the transducer array and the system capability. We summarize the challenges limiting the treatment envelope of this 360-element prototype system and propose potential strategies to alleviate them as follows.

*Challenge 1: The treatment envelope varies across patients due to different skull acoustic properties.*

Human skulls vary significantly in size, shape, thickness, sound speed, and cortical/trabecular heterogeneity. These factors produce large variations in trans-skull attenuation and the incident angles at which the acoustic beams from transducers collide with the skull surfaces. Phase aberration correction can compensate for the pressure loss due to aberration caused by variation in skull thickness and sound speed, but not for the losses due to scattering and reflection that are dependent on the skull shape and heterogeneity in skull microstructures. As a result, the acoustic power required to achieve effective treatment varies substantially across patients even when targeting at the same distance from the skull surface.

*Solution: Array design optimization based on a large skull dataset*

The 360-element transducer array used in this study was able to treat a wide range of locations in the skull with a high SDR and small thickness (skull#1) but did not have sufficient pressure headroom to treat shallow targets in skull #2 with a low SDR and large thickness. Due to the high attenuation, an array with high power is necessary to achieve sufficient pressure for histotripsy treatment skulls with low SDR and large thickness. This can be achieved by maximizing the array packing density with an optimized transducer distribution pattern (Rosnitskiy *et al* 2018) and increasing the pressure output from individual transducers by utilizing improved piezoelectric devices with higher transduction efficiency and pressure output capacities. Quantitative pressure maps based on simulations using a more extensive skull sample set would be helpful during the design process to determine reasonable pressure levels required for efficient and safe treatment in a broader group of patients.

*Challenge 2: Incident angles for shallow targets are much higher than at deep targets, resulting in significantly higher attenuation.*

Large incident angles were observed on the acoustic beams from more transducer elements when targeting shallow targets than central targets. For each element, the transmission rate through the skull decreases as the incident angle increases, both due to geometrical losses in the transmission ratio and increased attenuation losses as the pulses travel through longer paths within the bone itself. Furthermore, the high incident angles also pose challenges to aberration correction, as large incident angles have been shown to degrade the performance of ray-tracing models (Bancel *et al* 2021). When targeting within 10 mm from the skull surface, incident angles >25° were observed on more than 50% of the elements, hindering the efficacy of CT-based ray-tracing AC on those elements. The performance of 2-step AC was consequently impeded as CT-AC cannot recover sufficient focal pressure to initiate any cavitation for ACE-AC.

*Solution: Array pose optimization.*

Incident angles can be minimized by rotating the array to an optimized pose for each specific target location and patient, similar to the tcMRgFUS setup where the array can be rotated and translated mechanically around the patient’s head to place the target as close to the geometric focus of the array as possible (Meng *et al* 2020). The feasible range of rotation for a hemispherical array is limited to approximately ±15° on both the lateral and anterior-posterior axis around the patient’s head. Previously, a ray-tracing-based optimization model has been applied to predict the pressure increase by rotating the array pose (Gerhardson 2020). Our preliminary results suggested that optimizing the array pose can offer a 10%–20% focal pressure increase at shallow targets. The ray-tracing model is computationally efficient compared to the full-wave simulation approach used in this study but has lower accuracy as it does not account for scattering, absorption, and mode conversion through the skull. As the objective function for array pose optimization is nonconvex, an adequate solver should be identified and implemented in a computationally efficient way to produce solutions to the model in clinically appropriate time frames (<5 min). The hybrid angular spectrum (HAS) method has been shown to provide better accuracy than ray-tracing and substantially reduces the computation time compared to full-wave simulations (Leung *et al* 2021), (Almquist *et al* 2015, 2016), which may make it a good candidate for array pose optimization. Future work is necessary to explore the use of HAS to develop an adequate solver for this purpose and validate its performance experimentally.

*Challenge 3: Pre-focal cavitation at the pressure hotspot on the skull.*

Previous studies revealed that the porous microstructures in trabecular bone have strong variations in their acoustic properties and support both compressive and shear waves (Pinton *et al* 2012). Such heterogeneity on the sub-wavelength scale results in scattering, mode conversion, and multiple shear and compression resonance modes within the bone. When targeting near the skull surface, acoustic beams travel through longer path lengths in the skull, which leads to more significant scattering and mode conversion compared to deep targets and increases the probability of generating pre-focal pressure hotspots. These pre-focal hotspots prevent acoustic energy from being delivered efficiently at the desired target location and potentially induce damage in surrounding healthy tissue and bone marrow, thus limiting the treatment efficacy for shallow targets.

*Solution: Anti-focusing on the pre-focal pressure hotspot by amplitude correction*

Table 2 suggested that most pre-focal pressure hotpots can be eliminated or reduced significantly by performing phase aberration correction. As figure 8 demonstrates, most of the pre-focal hotspot pressure is attributable to a small number of transducer elements, which indicates that pre-focal hotspots may be reduced by de-activating or decreasing the transmission amplitude of these elements. As long as these elements do not completely overlap with the subset of elements that contribute most to focal pressure, the amplitude correction operation should still maintain sufficient pressure to generate on-target cavitation. For thermal-based FUS, anti-focusing approaches have been proposed to optimize the amplitude over the phased array by eliminating the strain tensor at a given position or introducing regularization terms when solving the inverse problem for re-focusing (Pulkkinen *et al* 2011), (Ebbini and Cain 1989), (Seo and Lee 2009). These techniques can reduce the heating at unwanted locations while maintaining sufficient acoustic power at the target. Alternatively, a binary mask can be applied to the transducer array to turn off the elements with high incident angles or in ‘no-pass zones’ near sensitive neurovascular structures. This technique is currently used in current clinical MRgFUS systems (Meng *et al* 2020). The performance of anti-focusing on the pre-focal pressure hotspot for transcranial histotripsy should be investigated by simulation and experiments in future studies.

This study demonstrates the capability of using a prototype transcranial histotripsy array to treat from deep to shallow locations in skulls with high SDR, however, the treatment location envelope is limited to the deep locations for skulls with low SDR. Therefore, this design is useful for a future clinical trial where the brain target is in deep locations or when treating selected patients is accepted. Our recent study has demonstrated volume ablations using the same prototype histotripsy system in three central brain regions (corpus callosum, septum pellucidum, and thalamus) in two cadavers (Choi *et al* 2024). If the clinical use requires both deep and shallow brain locations in a broad patient population, such as non-invasive surgery for brain pathologies that reside in the cerebral cortex, including brain tumors and ICH, a new transcranial histotripsy array needs to be re-designed and built based on the strategies described above.

In our experience, skull heating can be mitigated using a very low duty cycle (<0.1%) for deep central targets in selected skulls (Gerhardson *et al* 2017). Locations near the skull surface may require an even lower duty cycle than that for central locations, which may lengthen the treatment time. Future studies will be necessary to address the concern of skull heating for shallow targets and investigate the treatment parameters (e.g. reducing PRF/duty cycle) to minimize treatment time and off-target cavitation in transcranial histotripsy. The treatment outcomes of volume ablations in the shallow cerebral cortex by transcranial histotripsy will be carefully evaluated in future large animal models and human cadavers.

One limitation of this study is that the shear wave conversion was not modeled in simulation, which can be strong for shallow targets due to the large incident angles (Clement and Hynynen 2002). Literature has reported the shear transmission is much lower than the longitudinal transmission through the skull (<8% in the 500–900 kHz range) due to higher shear attenuation (White *et al* 2006). Therefore, we expect that the contribution of converted shear waves to focal pressure and pre-focal hotspots will be relatively small compared to the transmission of longitudinal pulses (Robertson *et al* 2017). Additionally, our simulation results for skulls #1& 2 in table 2 matched well with experimental measurements, suggesting that the longitudinal propagation was dominant for determining the focal pressure and thus the treatment envelope for transcranial histotripsy in these cases. Modeling shear wave conversion is unlikely to substantially alter the results of this study but will be implemented in future studies to investigate potential effects on the pre-focal pressure field during transcranial histotripsy. Challenges for modeling shear waves include increased computational complexity and estimating the shear sound speeds from CT, which will need to be addressed in future work.

## 5. Conclusion

This study evaluates the treatment location profile of a 750 kHz, 360-element hemispherical transducer array for transcranial histotripsy brain treatment. Treatment location profiles in 2 excised human skulls were experimentally characterized. Full-wave acoustic simulations were performed in 8 human skulls to analyze the ultrasound propagation at various target locations, as well as the pressure contribution by individual transducers for anatomical targets close to the skull surface. Results demonstrate the presented transcranial histotripsy array can treat from deep to shallow locations in skulls with high SDR, however, the treatment location envelope is limited to the deep locations for skulls with low SDR. Key limitations are identified, and strategies are proposed correspondingly for the next-generation transcranial histotripsy system to expand the treatment envelope. These findings are important to inform the transcranial histotripsy array design for future clinical trials given the treatment location needs of the brain pathology indication.

## Data Availability

The data cannot be made publicly available upon publication because no suitable repository exists for hosting data in this field of study. The data that support the findings of this study are available upon reasonable request from the authors.
